# How Can Qualitative Methods Be Applied to Behavior Analytic Research: A Discussion and Suggestions for Implementation

**DOI:** 10.1007/s40617-024-00917-1

**Published:** 2024-02-28

**Authors:** Victoria Burney, Angela Arnold-Saritepe, Clare M. McCann

**Affiliations:** School of Psychology, University of Auckland, Auckland, New Zealand

**Keywords:** Qualitative methodology, Behavior analysis, Research methods, Social validity

## Abstract

Behavior analysts in research and clinical practice are interested in an ever-expanding array of topics. They are compelled to explore the social validity of the interventions they propose and the findings they generate. As the field moves in these important directions, qualitative methods are becoming increasingly relevant. Representing a departure from small-n design favored by behavior analysts, qualitative approaches provide analysts a unique set of tools to answer questions that prioritize voice, experience, and understandings in context. Despite recognition of the value of qualitative approaches in other disciplines, application of qualitative methods in behavior analysis remains limited. One likely explanation is that behavior analysts are not yet fluent in applying qualitative approaches within their clinical and research investigations. To address this issue, exploration of qualitative research approaches in behavior analytic literature is needed, alongside practical advice for analysts who are interested in using qualitative methods. This article briefly outlines qualitative literature which pertains to behavior analysts wanting to incorporate qualitative methods into their inquiries. Attention is primarily drawn to the need for coherence in designing and implementing a robust qualitative study that aligns with the behavior analyst’s aims and perspective on knowing. A set of guiding questions are provided to orient behavior analysts to considerations in qualitative research and outline how analysts can conceptualize a strong qualitative study. This article aims to support increased application of qualitative methods by behavior analysts, where these methods best address the function of the behavior analytic investigation.

Since the inception of the field, behavior analysts have consistently used small-n design to demonstrate the effectiveness of behavior change interventions (Smith & Little, 2018). By precisely comparing an organism’s performance against its own baseline responding (Kazdin, 2011), small-n designs facilitate behavior analysts asking and answering questions such as “what happens to an individual’s responding when this intervention is applied?” and “how effective is this intervention at achieving measurable behavior change?”

Behavior analysts have increasingly recognized the place of meta-aggregation of small-n data and randomized control trials (Dowdy et al., 2021) to justify further-reaching generalizations, and appeal to growing audiences for behavior analytic research (Friman, 2021; Luiselli et al., 2023). Such methodologies have helped analysts address questions such as “how effective is this intervention for addressing the needs of a specific group?” and “how feasible is this intervention within large scale delivery?”

More recently, questions around the social validity of behavior analytic endeavors (including the social significance of goals, social appropriateness of procedures, and the social importance of outcomes; Wolf, 1978), as well as burgeoning interest in novel, or less well-defined, topics for study (including explorations of equity and social movements; Critchfield & Reed, 2017) have led behavior analysts to consider additional methodological tools for investigation of topics relevant to the field and wider world (Heward et al., 2022). Qualitative approaches have been identified as useful for behavior analysts asking questions focused on experiences, perspectives, and viewpoints (Burney et al., 2023), such as “how do stakeholders experience school based behavioral supports?” and “what functions are involved in determining effective climate change responses?”

A handful of recent publications exemplify the incorporation of qualitative approaches into behavior analytic literature. Anderson et al. (2022) used qualitative data generated through semi-structured parent interviews to inform development of social validity measures for behavior analytic feeding interventions. Pacia et al. (2022) incorporated qualitative data from surveys and group workshops with BCBAs into a mixed methods approach, refining a tool for working with parents (PAIRS). In a recent study, Leif et al. (2023) utilized open-ended survey responses and thematic analysis to understand experiences of behavior support practitioners working to reduce use of restrictive practices, including restraint and seclusion. Although representing a nonsystematically selected subset of research at the intersection of behavior analysis and qualitative research, these studies provide examples of how qualitative approaches can generate findings around social validity and stakeholder perspectives, to inform behavior analytic practice.

Given the commitment of behavior analysts to function informed clinical interventions (Ala’i-Rosales et al., 2019), it follows that behavior analysts should take an analogous approach to their empirical work. Such an approach would consider the aim of the research and requirements to answer the research question (i.e., function of the research), when selecting a methodological strategy, rather than fitting an empirical question to approaches the analyst is most familiar with (Malagodi, 1986). In doing so, behavior analysts could move away from “methodolatry” (or deference to a specific methodology based on its prominence; Chamberlain, 2000, p. 286) toward utilizing qualitative methods when research questions warrant this approach (Neuringer, 1991; Rohleder & Lyons, 2017).

Despite extensive use of qualitative methodologies within broader psychological enquiry (Flick, 2022; Levitt et al., 2017), the application of qualitative methods in behavior analysis is far from widespread. One plausible explanation is that analysts have a limited familiarity with, or fluency applying, qualitative methods in their work. Qualitative approaches are not routinely taught to trainee behavior analysts or rehearsed readily to allow for adoption into practice (Zayac et al., 2023). Taking the position that qualitative approaches offer behavior analysts an additional research tool, which can be functionally applied to questions where there is good fit, it follows that an introductory discussion of qualitative approaches, and how they might be sensitively incorporated into our scholarship, is warranted or possibly overdue.

This article aims to briefly outline qualitative research methodology, discuss how qualitative methods might relate to behavior analytic investigations, and pose a set of questions for behavior analysts to consider when attempting to incorporate qualitative methods into their clinical investigations or research activities. Attention is paid to how qualitative investigations can demonstrate rigor and quality, to be described as technological and analytic, and to how these investigations can be successfully adapted to feature in behavior analytic publications.

## What is Qualitative Methodology?

Qualitative research comprises “methodical scientific practices aimed at producing knowledge about the nature of experience and/or action” (Levitt et al., 2017, pp. 2–3), using “natural language and other descriptive and interpretive forms of human expression in their data, analysis, and findings” (Levitt et al., 2017, p. 3). Put simply, qualitative methods involve identifying meaning and interpreting patterns of experience using words, or textual responses, as data (Braun & Clarke, 2006) allowing people to share their thoughts and beliefs on their own terms (Avis, 2005). Far from a consistent “homogenous entity” (Smith, 2015), qualitative research has come to be considered an umbrella term for an overarching approach to empirical inquiry (Braun & Clarke, 2013). In this way, qualitative methodology is considered both an overarching paradigm for research, and a collection of specific techniques and approaches to doing research (Braun & Clarke, 2013; Smith, 2015).

In considering what constitutes a qualitative paradigm, Silverman and Marvasti (2008) propose the following elements: the use of naturally occurring data collection methods “that more closely resemble real life,” the analysis of words as data in ways that are “not reducible to numbers,” a preoccupation with meanings and making sense of phenomena as described by people, and a “rejection of the idea of the objective (unbiased) scientist” in favor of acknowledging the subjectivity of an investigator in shaping research outcomes (p. 8). Thus, qualitative research can be summarized as a focus on describing, rather than quantifying, people’s naturalistic reports of their own behavior or social phenomena, in order to interpret experiences of humans in context, which may later inform experimental approaches (Ashworth, 2008).

## Where Can Qualitative Methods Be Useful in Behavior Analysis?

For a field that is facing increased criticism attributed to social validity (Ferguson et al., 2018; Leaf et al., 2021), qualitative research may provide avenues for exploration. In particular, narrative accounts gleaned from stakeholders of behavior analytic services (defined broadly to include clients, families, wider communities, and policy makers; Schwartz et al., 1995) may help to measure the acceptability of goals, methods, and outcomes, to better understand the social validity of behavior analysis for those affected by it (Snodgrass et al., 2021). By taking an inductive approach and avoiding a narrow focus on explicitly defined or measurable variables (Patton, 2002), qualitative methods may help behavior analysts to understand lived experiences of interventions, informing a move toward more acceptable and appropriate services as defined by stakeholders. A notable example of applying qualitative methods to studying social acceptability is offered by Castro-Hostetler et al. (2022). Authors used qualitative data collection methods, including interviews and focus groups, to survey the experiences of Latino families accessing behavioral supports for autistic children in the United States. Qualitative methods elucidated the impact of language and cultural values on families’ experiences, directly canvassing the appropriateness of interventions from the unique perspective of parents with specific cultural contexts.

Qualitative methods are also useful when beginning to investigate novel areas where interesting and relevant variables are not explicit (i.e., we do not know the salient variables to manipulate), measurement is unclear (i.e., we do not know what we need to collect data on to evaluate change), stakeholder-led exploration is important (i.e., we do not have membership in the target group to set investigative priorities), or within co-designed or co-constructed investigations (i.e., with stakeholders defining the boundaries of study in line with their worldview and needs; Locock & Boaz, 2019). In these situations, qualitative approaches offer behavior analysts tools to explore diverse or novel areas of interest at the intersection of social issues, political movements, or culture and diversity concerns (Čolić et al., 2021). Offering an example, Max and Lambright (2021) utilized qualitative methods to investigate low fidelity of implementation for analysts working within school districts. Recognizing that abundant small-n studies measuring fidelity in school-based ABA delivery had not identified the key issues or produced change, the authors harnessed direct verbal reports of functions of behavior in school settings to inform development of a behavior change approach.

## How Do Qualitative Approaches Align with Dimensions of Behavior Analysis?

Although markedly different to traditional behavior analytic approaches to investigation, which prioritize focus on observable, discrete behaviors through single-subject manipulations, qualitative methods satisfy some dimensions of behavior analysis (Baer et al., 1968) deemed critical for robust inquiry (see Burney et al., 2023, for additional discussion). In particular, qualitative research can be congruent with the applied, technical, and generalizable dimensions of behavior analysis.

Contemporary qualitative research is characterized by an applied lens toward exploring socially important phenomena and creating meaningful change (Thorne, 2016). Although qualitative scholars have not always been interested in applied questions (e.g., historical sociological accounts focused on theory generation; Avis, 2005), current qualitative researchers, particularly in fields of heath and psychology, are interested in what can be learned from people and applied to the betterment of their situation and the situation of others. Interest in exploring insider perspectives of why behavior occurs in the manner in which it is observed or recorded—to inform improvements in clinical practice and adjust outcome measures to better reflect context—are commitments of qualitative research to practical utility (Silverman & Marvasti, 2008).

Current qualitative scholars value technological accounts of investigative processes and outcomes, to bolster the reliability and credibility of the research findings (Yardley, 2015). Although qualitative approaches diverge from quantitative investigations in their focus on objectivity, replicability, and precise control over variables (Lincoln et al., 2011), both methods aim to provide a full account of what was done, how, and by whom, to allow readers to evaluate the effectiveness of the research and trustworthiness of the findings. Given that qualitative research does not aim to be replicable, as the subjective position of the investigator would preclude (Gioia, 2021), qualitative scholars now expect that a careful outline of decisions and actions made throughout the study is provided, such that others can gauge how conclusions were reached and the validity of such conclusions (Braun & Clarke, 2023).

Although other fields aspire to extrapolate findings from large data sets to explain even larger population groups, behavior analysis has no such aspirations (Smith & Little, 2018). Grounded in an understanding that demonstrations of effect for an organism may be generalizable to others that share relevant contextual variables, behavior analysts hold measured extensions of generalizability to heart (Friman, 2021). Qualitative research holds the same reservations around generalization, aiming to explain or understand the experience of a small number of individuals, to gain insight into a situation, event, or phenomena as experienced by those involved (Smith, 2015), and to make measured applications of generality to others in similar contexts (Gioia, 2021). Neither approach warrants straying too far from the data, yet both approaches aim to disseminate findings to those with similar characteristics who may benefit. As a result, both behavior analytic investigations and qualitative research projects reject preoccupation with “representativeness” or specific numbers of participants (Higginbottom, 2004), in order to generate meaningful and carefully generalizable findings.

## Conceptualizing a Credible Qualitative Investigation within Behavior Analysis

Owing to the breadth of approaches considered under the qualitative umbrella (Smith, 2015), and continuing debate within qualitative circles on a range of contemporary issues and hot topics (Holloway & Todres, 2003), behavior analysts will accept that there is no “right way” to conduct an effective qualitative study. Far from providing an agreed or validated task analysis for conducting qualitative research, the following section aims to orient behavior analysts to key areas for consideration when utilizing qualitative methods in their work. Table 1 outlines key areas of consideration in designing a qualitative study, with prompt questions for analysts and suggested avenues for further reading.

### What is Your Epistemology and Perspective on Knowledge?

With reference to empirical investigations, epistemology has been described as the “philosophical meta theories or paradigms” (Chamberlain, 2015, p. 10) that outline what constitutes knowledge and reality, and guide decisions about what is important to study. These assumptions about what knowledge is and what it is possible to know inform the belief system or worldview of the investigator (Guba & Lincoln, 1994), shaping the way investigators plan and deliver research (Braun & Clarke, 2022).

Chamberlain (2015) contests that despite their importance, epistemological perspectives are generally afforded minimal attention, and are routinely “taken for granted” (p. 9) in research reporting. Braun and Clarke (2023) take this further, concluding that researchers who are trained in quantitative methodologies under one “overarching paradigm” (p. 10) see little need to discuss their epistemological leanings. In particular, the dominance of a positivist, or more routinely post-positivist, epistemology (Grant & Giddings, 2002) with a focus on an observable “truth” available for researchers to objectively record and manipulate, precludes discussion of what epistemological position is employed or how this relates to the research at hand. This contention aligns with behavior analytic investigations, where since Skinner’s early conceptualizations of “knowing” in radical behaviorism (Skinner, 1957, 1974), minimal reference is given to epistemology in contemporary behavior analytic literature.

It can be argued that for behavior analysts, a consideration of epistemological paradigms is less valuable, or not reinforced, in developing or communicating our research. It may only be that when venturing into qualitative investigations, where assumptions on “knowing” (defined as behavior relations influenced by the knower’s learning history and present contexts; Dittrich, 2020; Morris, 1993; Ruiz, 1995) are more hotly debated, contested, and diverse (Marecek, 2003), that attention is closely paid, and the behavior of tacting a specific epistemology is reinforced.

Within qualitative scholarship, positioning yourself and your way of “knowing” is a critical part of generating credible, and coherent, research. Contemporary scholars argue that, as distinct from quantitative research with one clear epistemological position, qualitative approaches in their many and variable forms allow for a variety of epistemologies to be realized through research (interested readers should consult Holloway & Todres, 2003, for an outline of this argument). Far from offering an exhaustive list of the various epistemological perspectives held in qualitative research, Patton (2002) cites the messy nature of these perspectives, which are subject to constant revision. Notwithstanding, contextualized or interpretive views (i.e., that knowledge is a product of context and that multiple accounts of reality are possible, or expected; Avis, 2005), constructivist perspectives (i.e., that language is not a neutral conduit of knowledge but that knowing is subjectively constructed through the process of researcher involvement; Braun & Clarke, 2022) and critical theory (i.e., that knowledge is relative to power and control and is highly subjective depending on positionality; Vincent & O’Mahoney, 2018) lend themselves more readily to a qualitative approach to investigation than to a quantitative frame.

It is clear that no one epistemological perspective is best aligned with qualitative research (Avis, 2005). Rather, it is the responsibility of a conscientious behavior analyst who wishes to deliver a conceptually systematic qualitative study to find a type of qualitative research that makes sense in light of a behavior analytic, likely post-positivist, perspective on the world and what is possible to know. The epistemological position of the researcher will determine which qualitative methods have a conceptual fit, which research questions will be appropriate, which data collection approaches are meaningful, and what conclusions can be drawn from the investigation (Braun & Clarke, 2023).

### What Types of Research Questions Are a Good “Fit?”

In contrast to quantitatively framed questions, which focus on explanation, prediction, and control, qualitative questions aim to explore an issue or phenomenon by identifying viewpoints and making sense of events in context (Agee, 2009; Ponterotto, 2005). Summarized by Thorne (2016), qualitative questions look to “generate empirical knowledge about human phenomena for which depth and contextual understanding would be useful and for which measurement is inappropriate, premature, or potentially misleading” (p. 44). Qualitative investigations do not begin with a hypothesis or a presumed outcome to test (Richards, 2021). Rather, qualitative questions invite exploration of a critical subject (Creswell, 2007) through questions such as “what does that experience feel like?” and “what does this topic mean to you?”

Qualitative scholars caution that unlike in quantitative investigations, where every question holds equal weight in terms of methods that may answer them, some qualitative questions are grounded in a particular approach, and should be used within that approach to avoid incoherence in the study (Avis, 2005; Creswell, 2007). Patton (2002) explains that the textual form of a qualitative question, and particular written conventions, can signal a commitment to a particular qualitative tradition such as ethnography (“what is the culture of this group of people?”), phenomenology (“what is the meaning and essence of the lived experience of this phenomenon for a person or group?”), or narrative analysis (“what does this story, and how it is told, reveal about the world from which it came?”). Behavior analysts are cautioned to craft a qualitative question that aligns with their chosen qualitative approach.

Alongside bolstering the credibility of the investigation, careful development of qualitative questions will benefit researchers in other ways. Primarily, defining the research question will help behavior analysts to evaluate if the question is in fact qualitative in nature, or if indeed the question should be rewritten to apply a quantitative methodology. Defining a strong qualitative question may also help analysts to narrow the scope and breath of the investigation to what is manageable, given available resources (Thorne, 2018). Further, a well-developed qualitative question will help to inform a behavior analyst’s selection of data collection methods and analysis tools, to ensure that the aim of the research and positionality align with the question (Flick, 2022). It is important to remember that developing a robust qualitative research question can support researchers in deciding which participants, and how many of them, to include in the study. Moving away from a quantitative aspiration for representative samples (Agee, 2009), clear research questions will assist in selecting participants who will generate useful insights about the phenomena under study, and support recruiting just enough participants to convincingly describe the phenomena or experience for the target group (Marecek, 2003).

### What Counts as Data?

Qualitative data types are broadly textual: an account of a phenomenon, experience, or event from the perspective of someone involved (Lyons, 2015). This aligns with use of verbal report as data which, although possibly less preferred and certainly more contentious than observational data or permanent product (Neuringer, 1991), is frequently utilized in quantitative behavior analytic investigations (Hayes & Wilson, 1993; Luque & O’Hora, 2016). This is particularly true where the phenomena of interest is how people explain or understand an event or situation, compared with direct verbal reports of corresponding observable responses (Malagodi, 1986; Ruiz, 1995).

Individual interviews overwhelmingly form the dominant method of qualitative data collection (Lyons, 2015; Patton, 2002). But this method is not without variability, incorporating structured interview schedules where questions are delivered with fidelity across participants (aligned with post-positivist approaches and not far removed from surveys or questionnaires) as well as open interviews without a schedule of questioning. Group interviews, focus groups, and dyad interviews are also common methods, as are repeated interviews with participants across time (Patton, 2002). Other forms of data collection in qualitative investigations are similar to those applied in traditional behavior analytic research, such as observations (structured, unstructured, video), field notes, and review of permanent textual products (clinical notes or medical records; Lyons, 2015). Yet others are more novel: review of diary entries, social media posts, chat room discussions, reflexive interviews, and use of a permanent product, such as photo or video, to generate discussion (Braun & Clarke, 2022).

Again, the specific form of data collected within a qualitative investigation and the specific methodological tools used to collect these data (e.g., interviews, focus groups, observation, field notes, reflexive video) should closely align with the empirical positioning of the study, the research questions driving investigation, and the findings that will sufficiently address these questions (Levitt et al., 2017). Congruence between epistemological position, research aim, and specific investigative questions is considered paramount to selecting and enacting effective data collection in qualitative inquiry (Spencer et al., 2014).

### What Methodological and Analytical Options Exist?

Many distinct approaches to doing qualitative research exist, and the number of “branded” or unique qualitative methodologies continues to grow in response to changing needs and interests of researchers (Braun & Clarke, 2013; Rennie, 2012; Thorne et al., 1997). Having many “forms” of qualitative research to select from (Lincoln et al., 2011) can make it tricky to decipher exactly how to behave when faced with a question that is best addressed from a qualitative stance. Although an examination of the different types of qualitative investigation is outside the scope of this article, mention of some commonly cited approaches might support behavior analysts in further reading. Popular options include: interpretative phenomenological analysis, grounded theory, narrative analysis, conversation analysis, interpretive description, discourse analysis, critical and feminist approaches, and cooperative or participatory action approaches. This list is by no means exhaustive, but signals the burgeoning variability in qualitative methods.

Some approaches, such as grounded theory (Charmaz, 2006), phenomenology (for an overview see: Connelly, 2010), and narrative analysis (Murray & Sools, 2015) provide what Chamberlain (2000, 2011) calls “off the shelf methodologies” that specify particular procedures and analytic steps, offering a concrete approach to qualitative study with established “do’s” and “don’t’s.” Still others, such as interpretivist and critical approaches (Pilgrim, 2014; Thorne, 2016), allow for diversity of methodological and analytical options, taking more of an “a la carte” approach to methods than a “prix fixe” dining option. Regardless, the methodological approach selected for a qualitative study will have some impact on the specific analytic procedures employed by a researcher once data are satisfactorily collected. Again, a range of processes exist, which place varying emphasis on inductive or deductive approaches to analysis (Braun & Clarke, 2022), as well as varying levels of preserving (e.g., telling a coherent story) or breaking down (e.g., coding and recombining) data products (Sandelowski, 2000).

Thematic analysis offers options to behavior analysts developing skills in qualitative research, as an analytic tool that Braun and Clarke (2006, 2013) contend is closer to a theoretically independent research technique, or method, than other approaches. At its most rudimentary level, thematic analysis involves transcription of vocal verbal data, comprehensive coding of data segments, and development of themes as units of meaning that outline patterns in the data (Braun & Clarke, 2006). The approach has a number of subsets or variants, depending on the terminal goal of the project, varying on the spectrum of generally more positivist (e.g., codebook analysis, coding reliability; Boyatzis, 1998; Guest et al., 2012; template analysis; King et al., 2018; and matrix coding; Nadin & Cassell, 2004) to more reflexive and interpretivist approaches (e.g., reflexive thematic analysis; Braun & Clarke, 2021).

### How Can You Demonstrate Rigor and Quality?

As with other aspects of qualitative inquiry, discussions around what comprises a quality study are subject to much debate (Yardley, 2015). and considerations of how to measure quality are continually being revised and reworked. Qualitative scholars highlight the differences between what is considered quality in a qualitative investigation, and what quantitative scholars, such as behavior analysts, might consider signposts of quality (Bradbury-Jones et al., 2017; Braun & Clarke, 2023; Lyons, 2011).

One clear example of these differences is evident in the positioning of the investigator. Many qualitative approaches see the researcher as playing an active role in data collection and analysis as a *subjective* part of the investigation. Thus, specific strategies to reduce the influence, or bolster the *objectivity*, of the researcher are not appropriate (Barbour, 2001). Instead, approaches that show the researcher has identified, or positioned, their place in the research are used to signal transparency and quality (Yardley, 2000). Although quantitative approaches value reliability and replicability, this is rarely an aim of qualitative research. Instead, examples of quality in qualitative studies will acknowledge the limits to generalizability of the findings, given the context in which the study was developed, avoiding making overrepresentative claims of the findings.

Within the contested space of quality, a focus on establishing generic standards for good qualitative research has emerged (e.g., Elliott et al., 1999; Levitt et al., 2018; Tracy, 2010; Yardley, 2000). These checklists, or guidelines, represent a perspective that using an agreed set of criteria for qualitative research will assist researchers in conducting high quality investigations. However, even as quality guidelines for qualitative research have proliferated, there are competing calls to avoid using checklists as a set of rigid rules for judging qualitative research (Yardley, 2015) in what Barbour (2001) calls an “overzealous and uncritical” application of checklists for quality (p. 1115). Instead, many qualitative scholars call for careful application of quality markers, in the form of guidelines or checklists, while appreciating the specific context and rationale of the project, as well as its epistemological positioning.

Contemporary views of quality posit that good qualitative research is coherent, consistent, and avoids contradictions: from development, data collection, analysis approaches, all the way to the conclusions and extrapolation of findings (Braun & Clarke, 2023). Although checklist criteria may be useful in affirming ideas of quality or rigor, quality from this perspective involves formulating a credible study by having the theoretical perspective, aim and methods all “line up” or “stack together” in a sensible way. Essential here is the concept of coherence (or the degree to which a study is internally consistent, comprehensive, and persuasive as a whole; Yardley, 2015). This holds parallels with conceptual systematicity in behavior analysis, focused on establishing clear links between theoretical approach, research question, methodological approach, and interpretation of outcomes. Equally, transparency (or the provision of sufficient information to allow readers to understand the nature and extent of research decisions; Demuth, 2013), is akin to the technological element of behavior analysis, in outlining the steps and decisions that were made within a project to ensure critical consumers can formulate their own conclusions (Lincoln et al., 2011). Development of tools such as audit trails, fieldwork notes, and comprehensive memos of decision making, support qualitative investigators in demonstrating transparency and technological inquiry. Other approaches, such as member checking or consensus making strategies (Thomas, 2017), can bolster the robustness of findings within qualitative investigations. Given contention around evidencing quality in qualitative work, behavior analysts should consider prioritizing congruence across all elements of a study (e.g., conceptualization, design, and implementation) informed, but not constrained, by checklists of quality indicators.

### How Can You Convince Readers and Reviewers?

It is critical that any convincing application of qualitative methodology in behavior analytic investigations requires analysts to develop skills and knowledge in qualitative research. Such a focus on building competence may include: engaging in further reading, access to courses or manualized content, working alongside an experienced collaborator, or seeking out mentoring within a behavioral skills training framework. Analysts new to qualitative research could utilize these approaches to build relevant skill repertoires, such as interviewing skills, practical experience in coding and analysis, and familiarity with qualitative software options (Patton, 2002). Developing competence is arguably the first step to ensuring that any qualitative output in behavior analysis is of high quality, and well-received by audiences within and outside the field.

From here, attention can be directed to ways qualitative findings are communicated (Friman, 2021). Behavior analysts are fluent in writing and interpreting small-n design investigations. However, behavior analysts are generally less familiar with communicating qualitative research, which may pose a barrier to the dissemination of this work within behavior analytic publications (Nicolson et al., 2020). Further, challenges within the publication system may hamper dissemination of qualitative research. It should be noted that limited experience on behalf of reviewers and editors in critiquing qualitative work, coupled with restrictions around word limits and stylistic conventions in some publication outputs (Braun & Clarke, 2023), may serve to punish analysts who try to report on this type of investigation.

To effectively share qualitative work, behavior analysts need to develop skills and confidence with including subjective information in written outputs, signaling theoretical perspectives of the project, and positioning the researcher within the project for congruence. These adjustments in content and style require redefining what is typically considered effective writing for a behavioral audience and readily accepted in flagship behavioral journals (Critchfield & Farmer-Dougan, 2014; Friman, 2021). Addressing a potentially emerging skill set for reviewers and editors who are less familiar with qualitative approaches, analysts could use tools in their behavior analytic repertoires—such as modelling, shaping, and providing constructive feedback—to support the publication of good quality, robust qualitative investigations in spaces behavior analytic audiences are likely to access. The field could look to other instructive efforts, such as Braun and Clarke’s (2022) “20 recommendations” for editors and reviewers in evaluating qualitative submissions, to bolster competence in this area.

## Conclusion

With increasing attention paid to the social acceptability of behavior analytic interventions, alongside interest in an increasing array of topics, behavior analysts in research and clinical practice may want to expand their investigative “toolbelts” beyond small-n design, to apply qualitative approaches where these are most appropriate. Indeed, changing contexts affecting the field necessitate that behavior analysts demonstrate behavioral flexibility in matching investigative approaches to the aim, or function, of their investigation for greatest success.

This article summarizes qualitative approaches as they pertain to behavior analysis and offers suggestions for how analysts can begin to conceptualize, develop, and implement qualitative research. Far from a road map or task analysis, this discussion article is more accurately considered a starting point for positioning credible qualitative studies within behavior analysis. Qualitative methods exist as a diverse class of approaches, not all of which share a philosophical and epistemological “fit” with behavior analysis. Despite this, many qualitative approaches are consistent with a behaviorist philosophy, and can offer analysts complementary tools for conceptualizing questions, data collection, data analysis and generation of findings, to progress our field.

This article is a launching pad. Behavior analysts stimulated by this approach are urged to apply qualitative methods where they best fit a research agenda. Development of skill repertoires relating to qualitative research, such as collecting, coding, and analyzing qualitative data, can be achieved through further reading and collaboration with experts in qualitative approaches. Analysts are encouraged to disseminate qualitatively derived findings within behavior analytic publications, to act as exemplars which may shape the behavior of readers and reviewers. Further tutorials, which offer task analyses for utilizing specific qualitative methodologies, are clearly useful to behavior analysts at this time.

**Table 1:** Question prompts and further reading for behavior analysts conducting qualitative studies

Steps in qualitative study design	Prompt questions for behavior analysts	Suggested further reading
What is your epistemology and perspective on knowledge?	- What is your overall theory on knowledge and knowing? - Can you position yourself and the qualitative study you are planning within this worldview? - Does your epistemological stance “match” other choices you are making about the study (i.e., overall approach, methods, analytic tools)? - Is there an obvious “clash” between your qualitative approach and your philosophy of what can be known or researched?	Avis, 2005; Chamberlain, 2015; Pascale, 2011
What research questions are a good fit?	- Is your question qualitative in nature (or is it best answered by a quantitative methodology)? - Can your question be answered (i.e., is it too broad, or not possible to “know” based on your research strategy, recruitment, and sampling methods)? - Does the topography of your question suggest a particular qualitative “discipline” (and is this consistent with your planned methods)?	Agee, 2009; Patton, 2002; Thorne, 2016
What counts as data?	- How will you gather information to answer the research questions (i.e., who to involve and how)? - Will you use more than one type of data collection activity (and are these aligned with your overall approach)? If so, how will you combine data products to make meaning? - Can you justify your data collection choices, in light of your chosen qualitative approach?	Lyons, 2015; Smith, 2015
What methodological and analytical options exist?	- Does your preferred methodological approach have good fit with your epistemology? - Is a particular method of analysis specified by your methodological approach (or are there a range of available choices that offer good conceptual fit)? - Will the findings derived from this analysis answer your research question?	Chamberlain, 2011; Ponterotto, 2005; Rohleder & Lyons, 2017
How can you demonstrate rigor and quality?	- Do the various elements of the study link closely together (and can you evidence this)? - What data can you collect to document transparency around actions taken in the study? - Will you use a checklist to assess the quality of your study (is there a set of guidelines that fits with your approach)?	Braun & Clarke, 2023; Lincoln et al., 2011; Yardley, 2015
How can you convince readers and reviewers?	- Do you have sufficient knowledge, skills, and competence to undertake a qualitative investigation? - Can you access resources to develop your competence in qualitative methods (i.e., didactic materials, modelling from a skilled collaborator, opportunities for practice with feedback)?	Braun & Clarke, 2013; Camic et al., 2003; Sullivan & Forrester, 2018

## Data Availability

Data sharing is not applicable to this article and no datasets were generated or analyzed as part of this discussion article.

## References

[CR1] Agee J (2009). Developing qualitative research questions: A reflective process. International Journal of Qualitative Studies in Education.

[CR2] Ala’i-RosalesCihonCurrierFergusonLeafLeafMcEachinWeinkaufx SJHTDRJLJBRJSM (2019). The big four: Functional assessment research informs preventative behavior analysis. Behavior Analysis in Practice.

[CR3] Anderson R, Taylor S, Taylor T, Virues-Ortega J (2022). Thematic and textual analysis methods for developing social validity questionnaires in applied behavior analysis. Behavioral Interventions.

[CR4] Ashworth, P. (2008). Conceptual foundations of qualitative psychology. In J. A. Smith (Ed.), *Qualitative psychology: A practical guide to research methods* (3rd ed., pp. 4–25). Sage.

[CR5] Avis, M. (2005). Is there an epistemology for qualitative research? In I. Holloway (Ed.), *Qualitative Research in Health Care* (pp. 3–17). McGraw-Hill Education. 10.1002/9780470750841

[CR6] Baer DM, Wolf MM, Risley TR (1968). Some current dimensions of applied behavior analysis. Journal of Applied Behavior Analysis.

[CR7] Barbour, R. S. (2001). Checklists for improving rigour in qualitative research: A case of the tail wagging the dog? *BMJ: British Medical Journal*, *322*(7294), 1115–1117. 10.1136/bmj.322.7294.1115 10.1136/bmj.322.7294.1115 PMC1120242 11337448

[CR8] Boyatzis, R., E. (1998). *Transforming qualitative information: Thematic analysis and code development*. Sage.

[CR9] Bradbury-Jones C, Breckenridge J, Clark MT, Herber OR, Wagstaff C, Taylor J (2017). The state of qualitative research in health and social science literature: A focused mapping review and synthesis. International Journal of Social Research Methodology.

[CR10] Braun V, Clarke V (2006). Using thematic analysis in psychology. Qualitative Research in Psychology.

[CR11] Braun, V., & Clarke, V. (2013). *Successful qualitative research: A practical guide for beginners*. Sage.

[CR12] Braun V, Clarke V (2021). One size fits all? What counts as quality practice in (reflexive) thematic analysis?. Qualitative Research in Psychology.

[CR13] Braun, V., & Clarke, V. (2022). *Thematic analysis: A practical guide*. Sage. 10.53841/bpsqmip.2022.1.33.46

[CR14] Braun V, Clarke V (2023). Is thematic analysis used well in health psychology? A critical review of published research, with recommendations for quality practice and reporting. Health Psychology Review.

[CR15] Burney V, Arnold-Saritepe A, McCann CM (2023). Rethinking the place of qualitative methods in behavior analysis. Perspectives on Behavior Science.

[CR16] Camic, P. M., Rhodes, J. E., & Yardley, L. (2003). Naming the stars: Integrating qualitative methods into psychological research. In P. M. Camic, J. E. Rhodes, & L. Yardley (Eds.), *Qualitative research in psychology: Expanding perspectives in methodology and design* (pp. 3–15). American Psychological Association. 10.1037/10595-001

[CR17] Castro-Hostetler M, Kille I, Lopez LV, Contreras BP (2022). Understanding the role of cultural values in ABA service delivery: Perspectives from Latino families. Behavior & Social Issues.

[CR18] Chamberlain K (2000). Methodolatry and qualitative health research. Journal of Health Psychology.

[CR19] Chamberlain K (2011). Troubling methodology. Health Psychology Review.

[CR20] Chamberlain K, Rohleder P, Lyons A (2015). Epistemology and qualitative research. Qualitative research in clinical and health psychology.

[CR21] Charmaz K (2006). Constructing grounded theory: A practical guide through qualitative analysis. Sage.

[CR22] Čolić M, Araiba S, Lovelace TS, Dababnah S (2021). Black caregivers’ perspectives on racism in ASD services: Toward culturally responsive ABA practice. Behavior Analysis in Practice.

[CR23] Connelly LM (2010). What is phenomenology?. Medsurg Nursing.

[CR24] Creswell, J. W. (2007). *Qualitative inquiry and research design: Choosing among five approaches*. Sage.

[CR25] Critchfield TS, Farmer-Dougan VF (2014). Isolation from the mainstream: Recipe for an impoverished science. European Journal of Behavior Analysis.

[CR26] Critchfield TS, Reed DD (2017). The fuzzy concept of applied behavior analysis research. The Behavior Analyst.

[CR27] Demuth, C. (2013). Ensuring rigor in qualitative research within the field of cross-cultural psychology. In Y. Kashima, E. S. Kashima, & R. Beatson (Eds.), *Steering the cultural dynamics: Selected papers from the 2010 Congress of the International Association for Cross-Cultural Psychology*. Retrieved October 16, 2023, from https://scholarworks.gvsu.edu/iaccp_papers/109/

[CR28] Dittrich A (2020). Who has the last word? Radical behaviorism, science, and verbal behavior about verbal behavior. Perspectives on Behavior Science.

[CR29] Dowdy A, Peltier C, Tincani M, Schneider WJ, Hantula DA, Travers JC (2021). Meta-analyses and effect sizes in applied behavior analysis: A review and discussion. Journal of Applied Behavior Analysis.

[CR30] Elliott R, Fischer CT, Rennie DL (1999). Evolving guidelines for publication of qualitative research studies in psychology and related fields. British Journal of Clinical Psychology.

[CR31] Ferguson JL, Cihon JH, Leaf JB, Meter SM, McEachin J, Leaf R (2018). Assessment of social validity trends in the *Journal of Applied Behavior Analysis*. European Journal of Behavior Analysis.

[CR32] Flick, U. (2022). Setting the agenda: Roles of design(ing) in qualitative research. In *The Sage handbook of qualitative research design* (Vol. 1). Sage. 10.4135/9781529770278

[CR33] Friman PC (2021). There is no such thing as a bad boy: The circumstances view of problem behavior. Journal of Applied Behavior Analysis.

[CR34] Gioia D (2021). A systematic methodology for doing qualitative research. Journal of Applied Behavioral Science.

[CR35] Grant BM, Giddings LS (2002). Making sense of methodologies: A paradigm framework for the novice researcher. Contemporary Nurse.

[CR36] Guba EG, Lincoln YS, Denzin NK, Lincoln YS (1994). Competing paradigms in qualitative research. Handbook of qualitative research.

[CR37] Guest G, MacQueen K, Namey E (2012). Applied thematic analysis. Sage.

[CR38] Hayes SC, Wilson KG (1993). Some applied implications of a contemporary behavior-analytic account of verbal events. The Behavior Analyst.

[CR39] Heward WL, Critchfield TS, Reed DD, Detrich R, Kimball JW (2022). ABA from A to Z: Behavior science applied to 350 domains of socially significant behavior. Perspectives on Behavior Science.

[CR40] Higginbottom G (2004). Sampling issues in qualitative research. Nurse Researcher.

[CR41] Holloway I, Todres L (2003). The status of method: Flexibility, consistency and coherence. Qualitative Research.

[CR42] Kazdin, A. E. (2011). *Single-case research designs: Methods for clinical and applied settings* (2^nd^ ed.). Oxford University Press. 10.1080/07317107.2012.654458

[CR43] King, N., Brooks, J., & Tabari, S. (2018). Template analysis in business and management research. In M. Ciesielska & D. Jemielniak (Eds.), *Qualitative methodologies in organization studies: Vol. II: Methods and possibilities* (pp. 179–206). Springer International. 10.1007/978-3-319-65442-3_8

[CR44] Leaf JB, Cihon JH, Leaf R, McEachin J, Liu N, Russell N, Unumb L, Shapiro S, Khosrowshahi D (2021). Concerns about ABA-based intervention: An evaluation and recommendations. Journal of Autism & Developmental Disorders.

[CR45] Leif ES, Fox RA, Subban P, Sharma U (2023). “Stakeholders are almost always resistant”: Australian behaviour support practitioners’ perceptions of the barriers and enablers to reducing restrictive practices. International Journal of Developmental Disabilities.

[CR46] Levitt HM, Bamberg M, Creswell JW, Frost DM, Josselson R, Suárez-Orozco C (2018). Journal article reporting standards for qualitative primary, qualitative meta-analytic, and mixed methods research in psychology: The APA Publications and Communications Board task force report. American Psychologist.

[CR47] Levitt HM, Motulsky SL, Wertz FJ, Morrow SL, Ponterotto JG (2017). Recommendations for designing and reviewing qualitative research in psychology: Promoting methodological integrity. Qualitative Psychology.

[CR48] Lincoln, Y. S., Lynham, S. A, & Guba, E. G. (2011). Paradigmatic controversies, contradictions, and emerging confluences, revisited. In *The Sage handbook of qualitative research* (4th ed., Vol. 2, pp. 97–128). Sage.

[CR49] Locock L, Boaz A (2019). Drawing straight lines along blurred boundaries: Qualitative research, patient and public involvement in medical research, co-production and co-design. Evidence & Policy.

[CR50] Luiselli, J. K., Bird, F., Maguire, H., & Gardner, R. M. (2023). Conducting and disseminating research. In J. K. Luiselli (Ed.), *Applied behavior analysis advanced guidebook* (2nd ed., pp. 437–460). Academic Press. 10.1016/B978-0-323-99594-8.00017-9

[CR51] Luque FC, O’Hora D (2016). Verbal reports in the experimental analysis of behavior. International Journal of Psychology & Psychological Therapy.

[CR52] Lyons A (2011). Advancing and extending qualitative research in health psychology. Health Psychology Review.

[CR53] Lyons A, Rohleder P, Lyons A (2015). Approaches to collecting data. Qualitative Research in Clinical and Health Psychology.

[CR54] Malagodi EF (1986). On radicalizing behaviorism: A call for cultural analysis. The Behavior Analyst.

[CR55] Marecek, J. (2003). Dancing through minefields: Toward a qualitative stance in psychology. In P. M. Camic, J. E. Rhodes, & L. Yardley (Eds.), *Qualitative research in psychology: Expanding perspectives in methodology and design.* (pp. 49–69). American Psychological Association. 10.1037/10595-004

[CR56] Max C, Lambright N (2021). Board certified behavior analysts and school fidelity of applied behavior analysis services: Qualitative findings. International Journal of Developmental Disabilities.

[CR57] Morris EK (1993). Behavior analysis and mechanism: One is not the other. The Behavior Analyst.

[CR58] Murray, M., & Sools, A. (2015). Narrative research in clinical and health psychology. In P. Rohleder & A. Lyons (Eds.), *Qualitative research in clinical and health psychology* (pp. 133–154). Bloomsbury Publishing. 10.1007/978-1-137-29105-9_9

[CR59] Nadin, S., & Cassell, C. (2004). Using data matrices. In C. Cassell & G. Symon (Eds.), *Essential guide to qualitative methods in organizational research* (pp. 271–287). Sage. 10.4135/9781446280119

[CR60] Neuringer A (1991). Humble behaviorism. *The*. Behavior Analyst.

[CR61] Nicolson AC, Lazo-Pearson JF, Shandy J (2020). ABA finding its heart during a pandemic: An exploration in social validity. Behavior Analysis in Practice.

[CR62] Pacia C, Gunning C, McTiernan A, Holloway J (2022). Developing the Parent-Coaching Assessment, Individualization, and Response to Stressors (PAIRS) tool for behavior analysts. Journal of Autism & Developmental Disorders.

[CR63] Pascale, C.-M. (2011). Cartographies of knowledge: Exploring qualitative epistemologies. *Sage*. 10.4135/9781452230368

[CR64] Patton, M. Q. (2002). *Qualitative evaluation and research methods* (3rd ed.). Sage. 10.1177/1035719X0300300213

[CR65] Pilgrim D (2014). Some implications of critical realism for mental health research. Social Theory & Health.

[CR66] Ponterotto JG (2005). Qualitative research in counseling psychology: A primer on research paradigms and philosophy of science. Journal of Counseling Psychology.

[CR67] Rennie DL (2012). Qualitative research as methodical hermeneutics. Psychological Methods.

[CR68] Richards, L. (2021). *Handling qualitative data: A practical guide* (4th ed.). Sage.

[CR69] Rohleder, P., & Lyons, A. (2017). Introduction. In P. Rohleder & A. Lyons (Eds.), *Qualitative research in clinical and health psychology*. Bloomsbury. 10.1007/978-1-137-29105-9_1

[CR70] Ruiz, M. R. (1995). B. F. Skinner’s radical behaviorism: Historical misconstructions and grounds for feminist reconstructions. *Psychology of Women Quarterly*, *19*(2), 161–179. 10.1111/j.1471-6402.1995.tb00285.x

[CR71] Sandelowski M (2000). Whatever happened to qualitative description?. Research in Nursing & Health.

[CR72] Schwartz IS, Staub D, Gallucci C, Peck CA (1995). Blending qualitative and behavior analytic research methods to evaluate outcomes in inclusive schools. Journal of Behavioral Education.

[CR73] Silverman, D., & Marvasti, A. (2008). *Doing qualitative research: A comprehensive guide*. Sage.

[CR74] Skinner, B. F. (1957). *Verbal behavior*. Appleton-Century-Crofts.

[CR75] Skinner BF (1974). About behaviorism.

[CR76] Smith, J. (Ed.). (2015). *Qualitative psychology: A practical guide to research methods* (3rd ed.). Sage.

[CR77] Smith P, Little D (2018). Small is beautiful: In defense of the small-N design. Psychonomic Bulletin & Review.

[CR78] Snodgrass MR, Chung MY, Kretzer JM, Biggs EE (2021). Rigorous assessment of social validity: A scoping review of a 40-year conversation. Remedial & Special Education.

[CR79] Spencer, L., Ritchie, J., Lewis, J., & Dillon, L. (2014). *Quality in qualitative evaluation: A framework for assessing research evidence*. UK Cabinet Office. https://www.cebma.org/wp-content/uploads/Spencer-Quality-in-qualitative-evaluation.pdf

[CR80] Sullivan, C., & Forrester, M. (2018). *Doing qualitative research in psychology: A practical guide* (2nd ed.). Sage. 10.4135/9781473914209

[CR81] Thomas D (2017). Feedback from research participants: Are member checks useful in qualitative research?. Qualitative Research in Psychology.

[CR82] Thorne S (2016). Interpretive description. Routledge.

[CR83] Thorne S (2018). What can qualitative studies offer in a world where evidence drives decisions?. Asia-Pacific Journal of Oncology Nursing.

[CR84] Thorne S, Kirkham SR, MacDonald-Emes J (1997). Interpretive description: A noncategorical qualitative alternative for developing nursing knowledge. Research in Nursing & Health.

[CR85] Tracy SJ (2010). Qualitative quality: Eight “Big-Tent” criteria for excellent qualitative research. Qualitative Inquiry.

[CR86] Vincent, S., & O’Mahoney, J. (2018). Critical realism and qualitative research: An introductory overview. In C. Cassell, A. Cunliffe, & G. Grandy (Eds.), *The Sage handbook of qualitative business and management research methods* (pp. 201–216). Sage. 10.4135/9781526430212.n13

[CR87] Wolf MM (1978). Social validity: The case for subjective measurement or how applied behavior analysis is finding its heart. Journal of Applied Behavior Analysis.

[CR88] Yardley L (2000). Dilemmas in qualitative health research. Psychology & Health.

[CR89] Yardley, L. (2015). Demonstrating validity in qualitative psychology. In J. A. Smith (Ed.), *Qualitative psychology: A practical guide to research methods* (3rd ed., pp. 257–273). Sage.

[CR90] Zayac RM, Van Stratton JE, Paulk AL (2023). An assessment of the qualities and behaviors of exemplary practitioners: Perspectives from international and award-winning behavior analysts. European Journal of Behavior Analysis.

